# Identification of Loci Modulating the Cardiovascular and Skeletal Phenotypes of Marfan Syndrome in Mice

**DOI:** 10.1038/srep22426

**Published:** 2016-03-01

**Authors:** Gustavo R. Fernandes, Silvia M. G. Massironi, Lygia V. Pereira

**Affiliations:** 1 Department of Genetics and Evolutionary Biology – Institute of Biosciences, University of São Paulo, São Paulo, Brazil; 2 Department of Immunology – Institute of Biomedical Sciences, University of São Paulo, São Paulo, Brazil

## Abstract

Marfan syndrome (MFS) is an autosomal dominant disease of the connective tissue, affecting mostly the skeletal, ocular and cardiovascular systems, caused by mutations in the *FBN1* gene. The existence of modifier genes has been postulated based on the wide clinical variability of manifestations in patients, even among those with the same *FBN1* mutation. Although isogenic mouse models of the disease were fundamental in dissecting the molecular mechanism of pathogenesis, they do not address the effect of genetic background on the disease phenotype. Here, we use a new mouse model, mg^ΔloxPneo^, which presents different phenotype severity dependent on the genetic backgrounds, to identify genes involved in modulating MFS phenotype. F2 heterozygotes showed wide phenotypic variability, with no correlations between phenotypic severities of the different affected systems, indicating that each has its specific set of modifier genes. Individual analysis of the phenotypes, with SNP microarrays, identified two suggestive QTL each to the cardiovascular and skeletal, and one significant QTL to the skeletal phenotype. Epistatic interactions between the QTL account for 47.4% and 53.5% of variation in the skeletal and cardiovascular phenotypes, respectively. This is the first study that maps modifier loci for MFS, showing the complex genetic architecture underlying the disease.

Marfan syndrome (MFS, OMIM #154700) is an autosomal dominant disorder of the connective tissue characterized by skeletal, ocular, cardiovascular, skin and pulmonary manifestations^1^. The disease affects 1–2/10,000 individuals and is caused by mutations in the *FBN1* gene that encodes fibrillin-1, the major structural component of microfibrils (reviewed in^2^). Although it is still not clear whether *FBN1* mutations lead to disease due to a dominant negative effect and/or to haploinsufficiency^3^, it is well established that fibrillin-1 containing microfibrils control the bioavailability of active TGF-β in the matrix, and that *FBN1* mutations lead to pathologically increased TFG-β signaling^4^. In fact, inhibition of TGF-β signaling in mouse models of MFS prevents the development of pulmonary and cardiovascular phenotypes, regardless of the presence of mutant fibrillin-1^5^.

Despite its complete penetrance, one trademark of MFS is its wide clinical variability^6^, where even siblings with the same mutation can display different age of onset and/or disease severity. The diversity of manifestations of MFS and lack of identifiable phenotype-genotype correlations suggest the existence of modifier genes^7^. Indeed, given the complex molecular pathogenesis of MFS and its pleiotropy, polymorphisms in a number of genes may modulate the effect of *FBN1* mutations in the different affected systems.

In 2010, Lima *et al.* reported the mg∆^loxPneo^ mouse model of MFS that develops skeletal, cardiovascular, and pulmonary alterations with different severities and age of onset between the two isogenic strains 129/Sv (129) and C57BL/6 (B6). These spectra of disease manifestations indicate that allelic differences between the two strains modulate MFS phenotype in a fashion more similar to human MFS than past isogenic murine models of the disease^8^.

We used the mg∆^loxPneo^ model to map loci associated with phenotype severity in MFS. By analysis of F1 and F2 crossed between B6 and 129 heterozygous for the *Fbn1* mutation, we show that each affected system has its own set of modifier genes. Moreover, we identify two quantitative trait loci (QTL) with suggestive linkages to the cardiovascular and skeletal phenotypes each, and one QTL with significant linkage to the skeletal phenotype, and show epistatic interactions among them.

## Material and Methods

### Animals

All animals were housed under controlled temperature and light conditions in a pathogen-free environment at the Immunology Department of the Instituto de Biociências at the University of São Paulo experimentation housing facility. The mapping population comprised 82 3-month-old 129 × B6 F2 heterozygous animals produced by crossing a wild-type B6 male and a heterozygous 129 female to generate F1 animals, then crossing wild-type and heterozygous F1 animals. From the F2 generation, a set of 46 animals exhibiting phenotypic extremes (skeletal, cardiovascular, or pulmonary manifestation) was obtained. This F2 approach is preferable to a backcross because it can identify interactions between loci and their effects on phenotype regardless of genotype, and it requires fewer animals than the backcross approach. To characterize how the phenotypes behave in a mixed background, every animal used to generate the F2 129 × B6 progeny had their phenotypes quantified three months after birth, except those selected for breeding. All animal experiments were approved by and conducted in accordance to the guidelines of the Institutional Animal Care and Use Committee of the Instituto de Biociências at the University of São Paulo.

### Fbn1^mgΔloxPneo^ allele genotyping

DNA was extracted from a 0.5-cm piece of tail using Proteinase K (Promega) as described by Zangala *et al.*^9^. Each sample underwent two independent PCR amplifications to identify the presence of the *Fbn1*^*mgΔloxPneo*^ allele and the normal allele, which served as an internal reaction control. *Fbn1*^*mgΔloxPneo*^ allele primers were as follows: forward 5′–GAG GCT ATT CGG CTA TGA CT–3′, reverse 5′–CTC TTC GTC CAG ATC ATC CT–3′. Cycling conditions were 94 °C for 2.5 min, then 30 cycles of 94 °C, 57 °C, and 72 °C for 1 min each in a 10 μl volume. *Fbn1*^*wt*^ allele primers were as follows: forward 5′–AAA CCA TCA AGG GCA CTT GC–3′, reverse 5′–CAC ATT GCG TGC CTT TAA TTC–3′. Cycling conditions were 94 °C for 2.5 min, then 30 cycles of 94 °C, 55 °C, and 72 °C for 1 min each in a 10 μl volume.

### Histological analysis

Animals were sacrificed by cervical dislocation. Mouse tissues were processed as previously described by Andrikopoulos *et al.*^10^. Five-micron sections were stained with hematoxylin and eosin, and adjacent sections were assayed for Weigert coloration, which is specific to elastic fiber visualization. Slides were examined and photographed using an Axiovert 200 (Carl Zeiss).

### Quantifying phenotypes

Skeletal (KR phenotype): A full body x-ray of each mouse was digitized and cervical-thoracic segment length and the straight-line distance of the same segment were measured using AutoCAD version 18.2. These measurements established a kyphosis ratio (segment length/straight distance; KR), which we used to score the severity of the skeletal manifestation of MFS. The smaller the ratio, the more severe the phenotype.

Cardiovascular (AWT phenotype): Histological samples were photographed at 50X and 100X magnification, and the lengths of the inner and outer perimeters of the aorta were measured using ImageJ^11^. From these data, we estimated the aortic wall thickness (AWT) for the inner and outer radius and wall of the aorta.

Pulmonary (Lm phenotype): The size of alveolar airways was determined by measuring the mean chord length on H&E-stained lungs as previously described^12^ This measurement is similar to the mean linear intercept (Lm), a standard measure of air-space size.

### Selective genotyping

From the 82 animals we selected 10 animals with extreme phenotypes to represent each tail of each phenotypic distributions, a total of 46 different animals, were genotyped with 7851 SNP microarrays.

SNP genotyping was conducted using the Illumina Infinium Mouse Genotyping microarray chip, and all procedures to determine the genotypes were established by Hellixa Company. An animal from each parental strain was genotyped together with the F2 animals as controls to identify the corresponding allele and informative SNPs.

### Synteny analysis

The synteny analysis was carried out using the Mouse Map Converter application^13^, to convert the QTL regions (cM) to physical distance intervals (bp), and the SyntenyTracker^14^ to identify the homology blocks between the mouse and human genomes. The existence of human homologues associated with diseases affecting MFS-related organ systems was verified with the Human-Mouse: Disease Connection tool available in the Mouse Genome Database^15^.

### Statistical Analysis

All statistical analysis were conducted in R version 2.12, with significance set at p = 0.05. The R/QTL package was used to identify genomic regions in linkage disequilibrium with each phenotype; markers that did not conform to Hardy-Weinberg equilibrium were removed from the analysis. Missing genotypes and pseudo-markers created every 1 cM were estimated using 1024 imputations. The suggestiveness (p < 0.63) and significance threshold (p < 0.05) were defined by 1024 stratified permutations due to the selective genotyping used to generate the data. The confidence intervals for each QTL were estimated using a Bayesian method as implemented in the R/QTL package^16^,^17^.

## Results

### Phenotypic characterization

**Figure 1:**
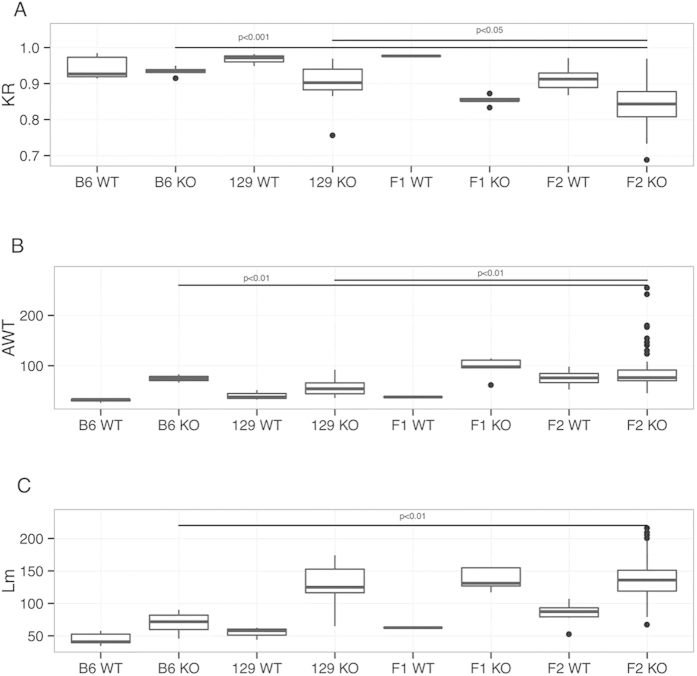
Quantification of phenotypes in parental, F1 and F2 animals. (**A**) Kyphosis ratio (skeletal phenotype, KR), (**B**) aortic wall thickness (cardiovascular phenotype, AWT), and (**C**) mean linear intercept (lung phenotype, Lm).

The animals from parental strains behaved as described by Lima *et al.*^8^; all F1 heterozygous animals had little variation, and disease manifestations were less severe than the 129 parental strain across all phenotypes [(skeletal (KR), vascular (AWT), and pulmonary (Lm)] (Fig. 1). However, the F2 animals displayed high variation across phenotypes, and the effects of this variability were even more deleterious than in the 129 parental strain.

The mean values for the F2 alterations in KR and AWT phenotypes were statistically different from the parental strains, although the F2 Lm phenotype was not significantly different from the 129 strain (Fig. 1). These phenotypic differences indicate the presence of modifier genes, with both strains possessing alleles that increase or decrease the severity of the phenotypes.

**Figure 2:**
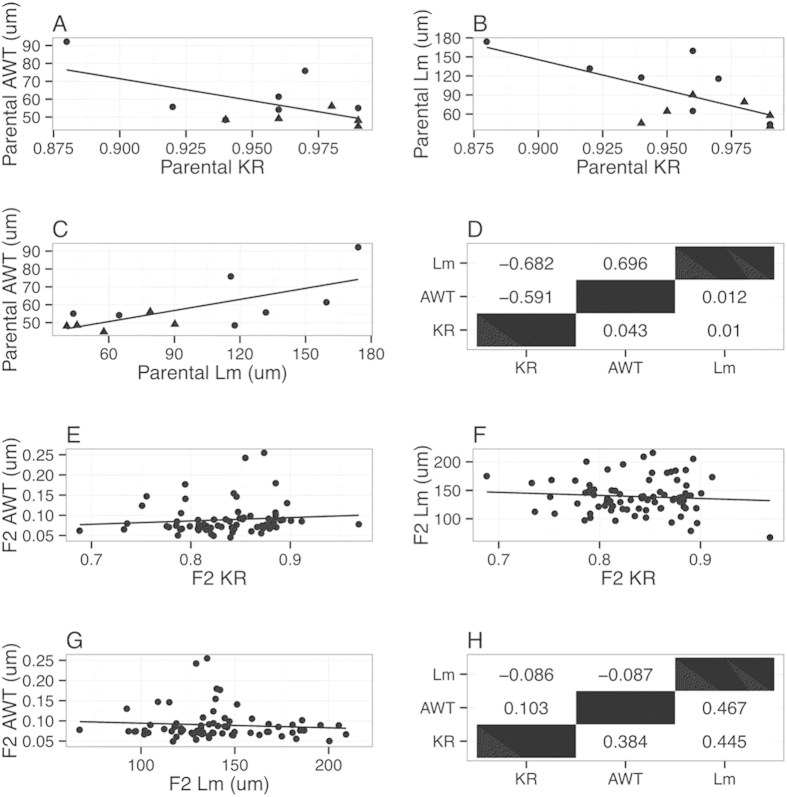
Correlation plots between the phenotypes for the parental and F2 animals. (**A–C**) Pairwise correlation plots between parental phenotypes (circles: 129 animals, triangles: B6 animals); (**D**) Summary of parental correlation (upper triangle) and p-values (bottom triangle); (**E–G**) Pairwise correlation plots between F2 phenotypes. (**H**) Summary of F2 correlation (upper triangle) and p-values (bottom triangle).

In the parental strains, there was concordance between phenotypic severity (Fig. 2A–D), with disease severity well-correlated among phenotypes. However, this concordance is not observed in the F2 animals (Fig. 2E–H), indicating that each phenotype has a unique set of modifier genes conferred by reassortment from the parental strains and, thus, genotypic studies (below) examined each phenotype individually.

Applying the heritability concept defined and formulated by Warner^18^ to the phenotypic distributions, we estimated that 40%, 76%, and 32% of the variability observed in the skeletal, cardiovascular, and lung manifestations, respectively, of the F2 animals was explained by genotype. The high heritability and the distributions of the phenotypes (S1 figure) suggest that they are more likely to be influenced by a few modifier genes, with each gene having large effects on phenotypic variation (the oligogenic model), than to be influenced by a large number of genes, each with a subtle phenotypic effect (polygenic model).

### Genetic Mapping

Missing data from the genotypes obtained from SNPs were filled based on imputations, and each phenotype was queried individually using a single-QTL model. Sex and age were tested for relevance as covariates for each model by ANOVA, but only sex achieved statistical significance as covariate and only for the AWT phenotype (p = 0.02).

**Figure 3:**
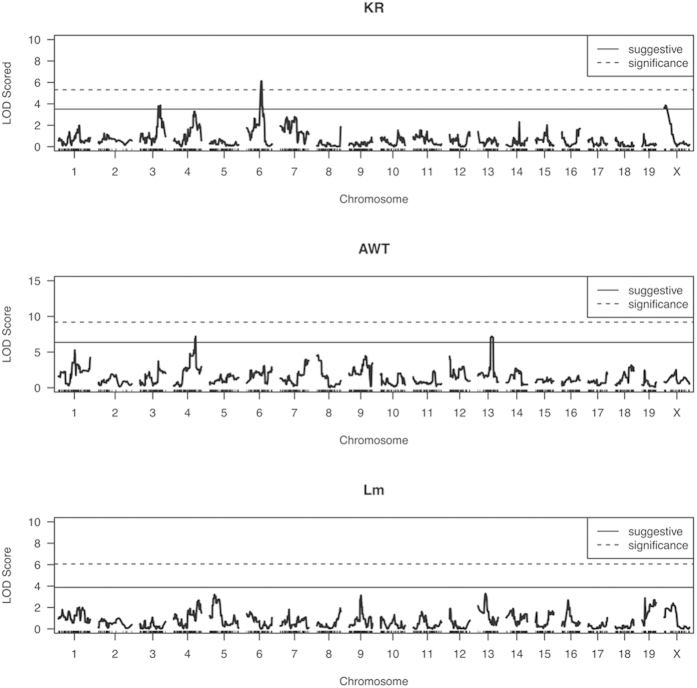
LOD score profile for each trait: (A) skeletal system (KR: kyphosis ratio), (B) cardiovascular (AWT: aortic root thickness), (C) pulmonary system (Lm: mean linear intercept). Significance threshold (p < 0.05) and suggestive threshold (p < 0.63) estimated using 1024 permutations.

For the KR phenotype, we identified evidence of significant linkage for a QTL on chromosome 6 (p < 0.05; LOD > 5.30) and two indications suggestive of linkage on chromosomes 3 and X (p < 0.63; LOD > 3.50) (Fig. 3, Table 1). Although we did not find any significant evidence of linkage for the AWT phenotype (p > 0.05; LOD > 9.19), there were two suggestive linkages on chromosomes 4 and 13 (p < 0.63; LOD > 6.35) (Fig. 3, Table 1). No linkages exceeding our threshold for suggestiveness were found in the Lm phenotype (Fig. 3).

### QTL effect upon trait

**Figure 4:**
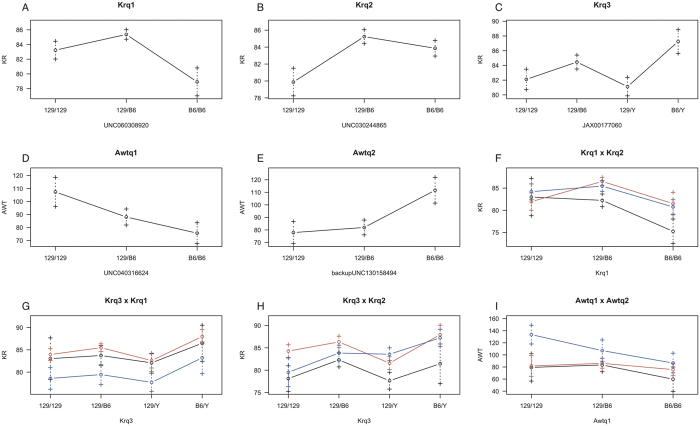
Effect plots for putative QTL Krq1(A); Krq2 (B); Krq3 (C); Awtq1 (D); Awtq2 (E); and interactions between them, Krq1 × Krq2 (F); Krq3 × Krq1 (G); Krq3 × Krq2 (H) and Awtq1 × Awtq2 (I). Genotypes of the SNPs closest to the estimated position of each QTL are plotted on the x-axis and by the line color for the second QTL in the interaction (black- 129/129, red- 129/B6, blue- B6/B6); quantification of the phenotypes is plotted on the y-axis. Values are expressed as mean ± standard deviation for each phenotypic class.

Based on the genotypes of the closest SNP to the estimated position of each QTL, we identified the dominance of QTL Krq1, Krq2, and Awtq2, with the effect of the 129 allele dominating the B6 allele in Krq1 and Awtq2, and B6 dominating 129 in Krq2 (Fig. 4); conversely, Awtq1 demonstrated an additive effect. The effect of Krq3 cannot be precisely identified without observing females homozygous for the B6 allele, which were not obtained from the crosses in this study.

We also identified epistatic effects on phenotypic traits. Krq1 and Krq2 interacted such that homozygosity for the B6 allele at Krq1 caused the KR phenotype to manifest in its most severe form only when a mouse was also homozygous for the 129 allele at the Krq2 locus (Fig. 4F). Awtq1 and Awtq2 also exhibited epistatic interactions: the additive behavior of Awtq1 can only be identified when the B6 allele is homozygous at the Awtq2 locus (Fig. 4I).

### Trait variability explained by QTL

Each of the QTL explains a portion of trait variability. To quantify that portion together with the additive and dominance effects, we established full regression models (with all QTL and interactions observed), and, based on an ANOVA, we identified the simplest model for both traits for which we identified putative QTL (KR and AWT phenotypes) (Table 2).

The full model for the KR phenotype consisted of Krq1, Krq2, Krq3, and the interaction between Krq1 and Krq2. This model explains 49.7% of the trait’s variability (p < 0.001); however, a second model omitting the Krq3 locus is not different from the full model, indicating that this term is not mathematically necessary to predict the KR trait. Thus, the final model consists only of Krq1, Krq2, and their interaction, explaining 47.4% of the trait’s variability (p < 0.001; Table 2).

For the AWT phenotype, the full model was composed of Awtq1, Awtq2, their interaction, the animal’s sex as a covariate, and the interaction of sex with both QTLs; this model explained 53.5% of the trait’s variability (p < 0.001). A simplified model that drops Awtq2 and all of its interactions is not significantly different from the full model, and can still explain 40.7% of the variability (p < 0.001; Table 2).

### Candidate Genes

Assuming that the point of largest linkage was the closest to the modifier gene and estimating the 95% confidence interval around this (Table 1), we produced a complete list of genes within each QTL (Supplementary Tables 1–5) and looked for those with interesting functions that could be correlated with the role of a modifier gene and, ultimately, phenotypic outcome. In particular, we searched for genes involved in the TGF-β signaling pathway, in protein processing and genes of extracellular matrix components (Table 3). Some of the identified genes were particularly relevant to the MFS phenotypes, including *Adamts9*, a member of the *Adamts* gene family involved in different diseases of connective tissue^19^; *Bmpr1b* and *Bmp15*, which encode type IB bone morphogenetic protein receptor and bone morphogenetic protein 15, respectively, both part of the TGF-β signaling pathway.

We next analyzed the regions of the human genome syntenic with the QTLs identified in mice (Table 4). While Krq3, Awtq1 and Awqt2 present one block of synteny each on human chromosomes X, 1 and 4, respectively, Krq1 and Krq2 are split in two synteny blocks each. Interestingly, Krq2 and Awtq1syntenic regions are approximately 9.5Mb apart on human chromosome 1, and therefore are likely to co-segregate in humans.

Human homologues for all the murine candidate genes localize within the synteny blocks. In addition, we identified 11 human genes involved in phenotypes associated with MFS, including different skeletal, cardiovascular and ocular abnormalities and (Table 4).

## Discussion

By including two mouse strains, B6 and 129, we were able to produce a murine model of MFS with wide variation in phenotypic severity in F2 animals, a scenario similar to the broad spectrum of human phenotypic severity among skeletal, pulmonary, and cardiovascular systems. Despite the use of a relatively small number of animals, we identified a significant linkage between the skeletal manifestation of MFS (KR phenotype) and a region of chromosome 6, Krq1. The interaction observed between a modifier gene on Krq1 and a possible modifier gene on Krq2 (chromosome 3) explained 47.4% of variability in the KR phenotype, indicating that both parental strains possess alleles affecting phenotypic severity that result in epistatic interactions in the F2 generation.

Our analyses suggest a linkage of the skeletal phenotype (KR) to the X chromosome, indicating a connection between phenotype and sex. Previous research has not described sex linkage of MFS or clinical severity; therefore, this finding is unusual and demands a closer look. Together with sex, modifiers on Awtq1 and Awtq2 (chromosomes 4 and 13, respectively) can explain 40.7% of the variability in the AWT phenotype. Although we cannot explain 33% of the variability, this uncertainty is related to the impossibility of detecting every gene involved in the phenotype, especially those with small effects upon the trait^20^ or that interact with loci in ways that have not been considered^21^,^22^,^23^. The lack of power to identify genes with small effects may be a reason to why there was no evidence of QTL linkage in the lung phenotype.

Although we were not able to narrow these proposed QTL regions sufficiently to describe the functional gene/SNP of each QTL affecting MFS phenotypes, this is the first study that maps modifier loci for MFS. Interestingly, genes with similar functions occur in all five QTL candidate regions, adding complexity to our knowledge of the MFS genetic architecture, given that functionally-similar changes can occur by several different routes and at different points within these routes.

Some of the gene families we identified in these QTL have already been associated with fibrillin-1/MFS, including the ADAMTS family, which is involved in extracellular matrix degradation and turn over, and a member of which, ADAMTS10, has been show to interact with fibrillin-1^24^; and genes in the TGF-β signaling pathway, the over-activity of which leads to MFS phenotypes^4^,^25^,^26^.

In addition, genes involved in protein processing may also be relevant for the MFS phenotype. Impaired secretion and intracellular retention of mutant fibrillin-1 have been show in MFS patients^27^,^28^. Similarly, the mutant *Fbn1*^mgΔloxPneo^ allele generates a truncated fibrillin-1 monomer that tends to accumulate inside the cell^8^. Thus, it is possible that polymorphisms in proteins involved in folding, exocytosis, or degradation, such as those of the chaperone class, may lead to improvement or exacerbation of MFS phenotypes^29^.

Finally, we identified additional candidate genes at the synthenic regions on the human genome based on their previously described connection to syndromes/phenotypes involving systems affected in MFS. In some instances, this involvement is secondary to a primary defect in an organ system unrelated to MFS – for example, in Nemaline Myopathy, skeletal abnormalities result from muscle weakness caused by a primary dysfunction in actin polymerization. Nevertheless, this indicates a role for the corresponding gene *LMOD3*, albeit indirect, for normal skeletal system function.

In conclusion, in this study, we identified five new loci involved in the modulation of MFS phenotypes. These findings represent the first mouse model to shed light on the complex genetic architecture underlying MFS variability. The complexity of the disease is such that each phenotype possesses a unique set of modifier genes with additive and epistatic effects upon each other and on the phenotype as a whole. We identified QTL explaining nearly half of the variability observed in the F2 animals for the KR phenotype and more than 40% of the AWT phenotype, indicating that there are more loci or interactions between loci that remain to be identified for both phenotypes.

The QTL identified here are restricted to these two mouse strains, and studies involving different strains might increase the understanding of how these QTL affect phenotypes under different environmental conditions, and what implications these QTL have for MFS as a whole. Identification of the specific genes involved in modulating the phenotype of the different affected systems will improve our understanding of the basic biology of the different organ systems involved in the disease. Furthermore, clarifying these QTL and the genes they contain should lead to an improved understanding of the molecular pathways involved in the development of each clinical manifestation, which, in turn, may lead to novel therapeutic strategies for MFS.

## Additional Information

**How to cite this article**: Fernandes, G. R. *et al.* Identification of Loci Modulating the Cardiovascular and Skeletal Phenotypes of Marfan Syndrome in Mice. *Sci. Rep.* **6**, 22426; doi: 10.1038/srep22426 (2016).

## Supplementary Material

Supplementary Information

## Figures and Tables

**Table 1:** Description of candidate quantitative trait loci (QTL) for phenotypic manifestations of MFS in the mg^ΔloxPneo^ mouse model.

Trait	Chr	QTL Name	Closest marker	Estimated position (cM)	95%-CI (cM)	LOD Score	Number of genes
KR	6	*Krq1*	UNC060308920	45.85	[41.0–49.1]	6.12	70
KR	3	*Krq2*	UNC030244865	64.49	[56.1–68.4]	3.85	139
KR	X	*Krq3*	JAX00177060	5.91	[2.4–20.2]	3.84	398
AWT	4	*Awtq1*	UNC04036624	68.74	[66.8–70.6]	7.19	150
AWT	13	*Awtq2*	backupUNC130158494	47.79	[44.4–52.1]	7.19	121

Chr: chromosome; CI: confidence interval.

**Table 2:** Results for the final model of multiple QTL, indicating the percentage of the variance explained by each QTL, covariate, or interaction.

Trait	Parameter	%var	Genetic Effect		P-value
			Additive	Dominancy	
KR	Intercept		84.15		
	Krq1	28.9	−6,84	2,15	1.12e-5
	Krq2	18.3	−2,53	−5,4	1.05e-3
	Krq1 × Krq2a	8.4	10.87	12.32	2.74e-2
	Krq1 × Krq2d		10.22	6.58	
AWT	Intercept		102.67		
	Awtq1	34.62	−63,15	−43,34	3.34e-6
	Sex	28.87	−22,28		7.9e-6
	Awtq1 × sex	22.88	62,4	60,34	2.09e-5

quantification of genetic effects (additive and dominant); p-value of the model without the term when compared against the full model.

**Table 3:** Candidate modifier genes identified within each QTL.

	Krq1	Krq2	Krq3	Awtq1	Awtq2
TGF-β pathway	*Foxp1*	*Bmpr1b*	*Bmp15Foxp3*		*Foxd1Zfyve16*
Protein processing	*Ube3Adamst9*	*Dnajb14*	*Usp27xUba1Porcn*		*Tbca*
Extracellular matrix		*Col25a1Npnt*		*Hspg2*	

**Table 4:** Synthenic regions and candidate modifier genes in the human genome.

QTL	Human Synteny Blocks position (bp)	Gene	human disease	Phenotype*	OMIM**
Krq1	chr3:238279-1291341; chr3:64079543-7457029	*EOGT*	Adams-Oliver syndrome	abnormality in skin development; malformations of the limbs;	615297
		*LMOD3*	Nemaline Myopathy 10	myopathy; feeding and swallowing difficulties; foot deformities; scoliosis; joint deformities	616165
Krq2	chr1:68564156-89458636; chr4:95373037-120550146	*LARP7*	Alazami syndrome	inscostant skeletal findings (scoliosis)	615071
Krq3	chrX:48316920-51151687	*CLCN5*	Dent disease	bone defects caused by faliure to reabsorb calcium	300009
		*PORCN*	focal dermal hypoplasia	skin, skeleton, eyes, and face	300651
Awtq1	chr1:895967-59012474	*ALPL*	Hypophosphatasia	bone and teeth malfomation (mineralization disruption)	146300; 241510; 241500
		*ECE1*	Hirschsprung disease cardiac defects and autonomic dysfunction	cardiac defects (ductus arteriosus, small subaortic ventricular septal defect, and small atrial septal defect), hypertension	613870
		*FUCA1*	fucosidosis	dysostosis multiplex (skeletal defects), respiratory infections	230000
		*HSPG2*	Schwartz-Jampel syndrome	skeletal phenotypes (kyphoscoliosis)	255800
Awtq2	chr5:43444354-96143803	*RASA1*	Parles-Weber syndrome	capillary malformations; arteriovenous fistulas; limb overgrowth	608355
		*VCAN*	Wagner syndrome	retinal detachment and cataract	143200

*phenotypes in MFS affected systems.

**entry number at the Online Mendelina Inheritance in Men database (www.omim.org).
