# Supplementary material for: Identification of Loci Modulating the Cardiovascular and Skeletal Phenotypes of Marfan Syndrome in Mice
Source: Sci Rep. 2016 Mar 1;6:22426. doi: 10.1038/srep22426 (PMC4772474; doi:10.1038/srep22426)
Supplement: Supplementary Information [file srep22426-s1.pdf]

IDENTIFICATION OF LOCI MODULATING THE CARDIOVASCULAR AND SKELETAL PHENOTYPES OF MARFAN SYNDROME IN MICE

Gustavo R. Fernandes[1], Silvia M. G. Massironi[2], Lygia V. Pereira[1*].

[1]Department of Genetics and Evolutionary Biology – Institute of Biosciences, [2]Department of Immunology – Institute of Biomedical Sciences, University of São Paulo, São Paulo, Brazil.

* corresponding author (lpereira@usp.br)

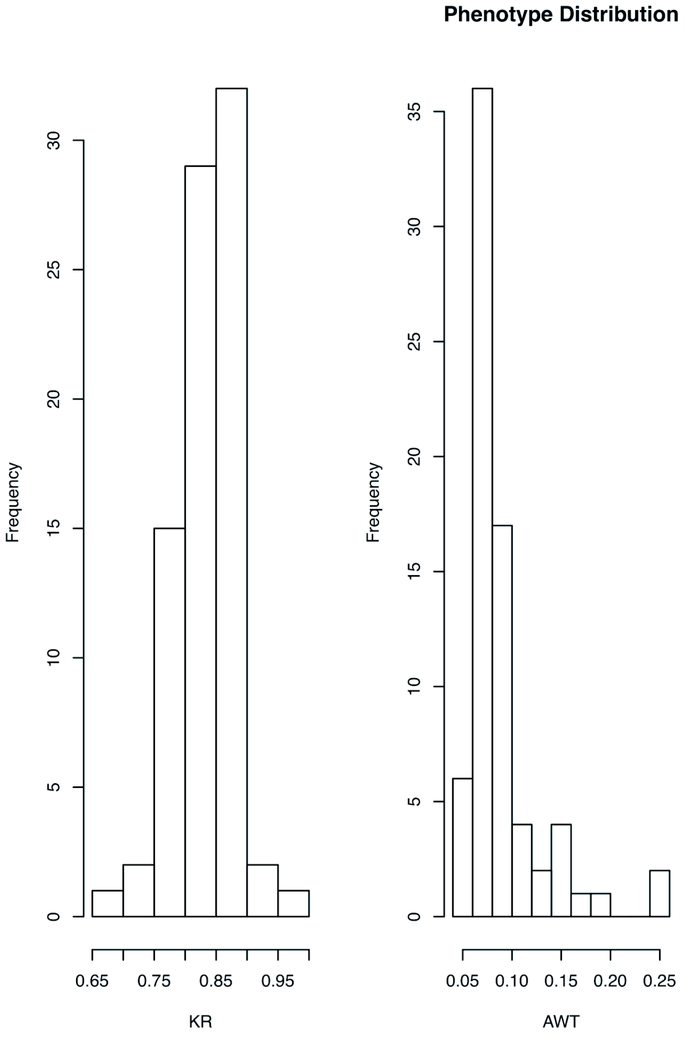

Supplementary Figure 1: Phenotypic distribution for the skeletal (KR), cardiovascular (AWT) and pulmonary (Lm) manifestations.

Supplementary Table 1: Genes in the Chromosome 6 locus Krq1.

1700010K10Rik	*1700123L14Rik*	*5_8S_rRNA*	*7SK*	*7SK*
A730049H05Rik	*AC131676.1*	*AC131700.1*	*AC152987.1*	*AC157096.1*
AC158665.1	*AC162038.1*	*Adamts9*	*Arl6ip5*	*AY512915*
Chl1	*Cntn3*	*Cntn4*	*Cntn6*	*Eif4e3*
Eogt	*Fam19a1*	*Fam19a4*	*Foxp1*	*Frmd4b*
Gm10234	*Gm15531*	*Gm15576*	*Gm15631*	*Gm15737*
Gm20696	*Gm20705*	*Gm6565*	*Gm765*	*Gm9871*
Gpr27	*Gxylt2*	*Kbtbd8*	*Lmod3*	*Lrig1*
Magi1	*Mitf*	*Pdzrn3*	*Ppp4r2*	*Prickle2*
Prok2	*Rybp*	*Shq1*	*Slc25a26*	*SNORA17*
SNORA17	*SNORA17*	*SNORA17*	*SNORA36*	*SNORA44*
SNORA48	*SNORA63*	*Suclg2*	*Tmf1*	*Tpt1-ps3*
Trh	*U1*	*U6*	*U6*	*U6*
U6	*U7*	*U7*	*Uba3*	*Zfyve20*

Supplementary Table 2: Genes in the Chromosome 3 locus Krq2.

1110002E22Rik	*4930422G04Rik*	*4930579F01Rik*	*5730508B09Rik*	*5S_rRNA*
AC098732.1	*AC099576.1*	*AC102128.1*	*AC111097.1*	*AC114668.1*
AC125040.1	*AC135118.1*	*AC140486.1*	*AC140486.2*	*AC164408.1*
AC165333.1	*Adh1*	*Adh4*	*Adh5*	*Adh6-ps1*
Adh6a	*Adh6b*	*Adh7*	*Agxt2l1*	*Aimp1*
Alpk1	*Ank2*	*Ap1ar*	*B930007M17Rik*	*Bank1*
Bdh2	*Bmpr1b*	*Casp6*	*Ccbl2*	*Ccdc109b*
Cenpe	*Cfi*	*Cisd2*	*Col25a1*	*Cxxc4*
Cyp2u1	*D630013G24Rik*	*Dapp1*	*Ddit4l*	*Dkk2*
Dnajb14	*Egf*	*Eif4e*	*Elovl6*	*Emcn*
Enpep	*Gar1*	*Gbp1*	*Gbp2*	*Gbp3*
Gbp5	*Gbp7*	*Gm10650*	*Gm15460*	*Gm15540*
Gm15688	*Gm15689*	*Gm16060*	*Gm16100*	*Gm16238*
Gm16559	*Gm2574*	*Gm4392*	*Gm4861*	*Gm4862*
Gm5105	*Gm5549*	*Gm9387*	*Gm9396*	*Gm9799*
Gstcd	*Gtf2b*	*H2afz*	*Hadh*	*Ints12*
Lamtor3	*Larp7*	*Lef1*	*Manba*	*Metap1*
Mir1895	*Mir1956*	*Mir302a*	*Mir302b*	*Mir302c*
Mir302d	*Mir367*	*Mttp*	*Neurog2*	*Nfkb1*
Nhedc1	*Nhedc2*	*Npnt*	*Oaz2-ps*	*Ostc*
Papss1	*Pdha2*	*Pdlim5*	*Pitx2*	*Pkn2*
Pla2g12a	*Ppa2*	*Ppp3ca*	*Rap1gds1*	*Rg9mtd2*
Rpl34	*Rrh*	*Sec24b*	*Sgms2*	*Slc39a8*
SNORA17	*SNORA17*	*SNORA17*	*SNORA17*	*SNORA32*
SNORA61	*Tacr3*	*Tbck*	*Tet2*	*Tifa*
Tspan5	*U1*	*U6*	*U6*	*U6*
U6	*U6*	*U6*	*U6*	*U6*
U6	*U6*	*Ube2d3*	*Unc5c*	

Supplementary Table 3: Genes in the X Chromosome locus Krq3.

1700012L04Rik	*1700054O13Rik*	*1810030O07Rik*	*2010204K13Rik*	*2900002K06Rik*
2900008C10Rik	*4930402K13Rik*	*4930453H23Rik*	*4930524L23Rik*	*4930557A04Rik*
4930578C19Rik	*4933416E14Rik*	*5730405O15Rik*	*5S_rRNA*	*5S_rRNA*
A230072C01Rik	*AA414768*	*Agtr2*	*Akap4*	*AL662925.1*
AL671117.1	*AL671211.1*	*AL671492.1*	*AL671885.1*	*AL671885.2*
AL671922.1	*AL732400.1*	*AL773580.1*	*Araf*	*Atp6ap2*
AU022751	*B630019K06Rik*	*BC049702*	*Bcor*	*Bmp15*
BX571729.1	*Cacna1f*	*Cask*	*Ccdc120*	*Ccdc22*
Ccnb3	*Cdk16*	*Cfp*	*Chst7*	*Clcn5*
Csnk2a1-ps	*CT867956.1*	*CT867960.1*	*CT868697.1*	*CT954250.1*
CT963124.1	*CT963124.2*	*CT963124.3*	*CU468592.1*	*Cybb*
Cypt1	*Ddx3x*	*Dgkk*	*Drr1*	*Dusp21*
Dynlt3	*E330010L02Rik*	*Ebp*	*Efhc2*	*Elk1*
Eras	*Foxp3*	*FQ976806.1*	*Fthl17*	*Ftsj1*
Fundc1	*Gata1*	*Glod5*	*Gm10058*	*Gm10096*
Gm10147	*Gm10230*	*Gm10487*	*Gm10488*	*Gm10490*
Gm10491	*Gm14363*	*Gm14365*	*Gm14366*	*Gm14367*
Gm14369	*Gm14370*	*Gm14373*	*Gm14374*	*Gm14375*
Gm14379	*Gm14380*	*Gm14381*	*Gm14394*	*Gm14395*
Gm14448	*Gm14450*	*Gm14451*	*Gm14452*	*Gm14457*
Gm14458	*Gm14459*	*Gm14470*	*Gm14473*	*Gm14474*
Gm14475	*Gm14476*	*Gm14477*	*Gm14478*	*Gm14479*
Gm14480	*Gm14481*	*Gm14482*	*Gm14483*	*Gm14484*
Gm14485	*Gm14491*	*Gm14493*	*Gm14499*	*Gm14500*
Gm14501	*Gm14502*	*Gm14503*	*Gm14504*	*Gm14505*
Gm14506	*Gm14509*	*Gm14510*	*Gm14511*	*Gm14512*
Gm14513	*Gm14514*	*Gm14515*	*Gm14516*	*Gm14517*
Gm14518	*Gm14519*	*Gm14520*	*Gm14521*	*Gm14523*
Gm14524	*Gm14525*	*Gm14527*	*Gm14529*	*Gm14530*
Gm14531	*Gm14533*	*Gm14534*	*Gm14535*	*Gm14536*
Gm14537	*Gm14538*	*Gm14539*	*Gm14558*	*Gm14561*
Gm14562	*Gm14563*	*Gm14632*	*Gm14634*	*Gm14635*
Gm14636	*Gm14703*	*Gm14720*	*Gm14799*	*Gm14820*
Gm14862	*Gm14863*	*Gm14864*	*Gm14865*	*Gm14867*
Gm14883	*Gm14884*	*Gm14885*	*Gm14974*	*Gm15027*
Gm15028	*Gm15029*	*Gm15030*	*Gm15276*	*Gm15280*
Gm15432	*Gm16265*	*Gm16479*	*Gm16480*	*Gm16481*
Gm1848	*Gm1989*	*Gm2003*	*Gm2005*	*Gm2012*
Gm2030	*Gm2066*	*Gm2092*	*Gm2101*	*Gm2117*
Gm2165	*Gm2200*	*Gm2759*	*Gm2768*	*Gm2777*

Gm2784	*Gm2790*	*Gm2799*	*Gm2825*	*Gm3657*
Gm3669	*Gm3677*	*Gm3681*	*Gm4297*	*Gm4732*
Gm4789	*Gm4836*	*Gm4906*	*Gm4907*	*Gm4984*
Gm4985	*Gm5073*	*Gm5123*	*Gm5124*	*Gm5132*
Gm5168	*Gm5169*	*Gm5379*	*Gm5380*	*Gm5381*
Gm5382	*Gm5383*	*Gm5384*	*Gm5634*	*Gm5635*
Gm5751	*Gm5752*	*Gm5753*	*Gm5754*	*Gm5755*
Gm5924	*Gm5926*	*Gm5926*	*Gm5931*	*Gm5934*
Gm5935	*Gm6071*	*Gm6079*	*Gm6113*	*Gm6121*
Gm6592	*Gm6787*	*Gm6797*	*Gm6798*	*Gm6826*
Gm6829	*Gm6923*	*Gm6938*	*Gm6956*	*Gm7079*
Gm7129	*Gm7164*	*Gm7350*	*Gm7365*	*Gm7375*
Gm7386	*Gm7391*	*Gm7398*	*Gm7408*	*Gm7415*
Gm7421	*Gm7437*	*Gm7438*	*Gm9083*	*Gm9085*
Gm9428	*Gm9429*	*Gm9430*	*Gm9431*	*Gm9432*
Gm9434	*Gm9435*	*Gm9436*	*Gm9439*	*Gm9440*
Gmcl1l	*Gpkow*	*Gpr34*	*Gpr82*	*Gripap1*
Hdac6	*Kcnd1*	*Kdm6a*	*Klhl13*	*Lancl3*
Magix	*Maoa*	*Maob*	*Med14*	*Mid1ip1*
Mir1198	*Mir188*	*Mir221*	*Mir222*	*Mir362*
Mir500	*Mir501*	*Mir532*	*Mycs*	*n-R5s2*
n-R5s3	*n-R5s5*	*Ndp*	*Ndufb11*	*Nudt10*
Nudt11	*Nyx*	*Otc*	*Otud5*	*Pcsk1n*
Phf16	*Pim2*	*Plp2*	*Porcn*	*Ppp1r2-ps7*
Ppp1r2-ps9	*Ppp1r3f*	*Pqbp1*	*Praf2*	*Prickle3*
Psmb6-ps	*Psmb7-ps2*	*Rbm10*	*Rbm3*	*Rgn*
RP23-367O24.1	*Rp2h*	*Rpgr*	*Rpl19-ps12*	*Rpl22-ps1*
Rpl23a-ps13	*Rpl3-ps1*	*Rpl30-ps11*	*Rpl36-ps10*	*Shroom4*
Slc35a2	*Slc38a5*	*Slc6a14*	*Slc9a7*	*Slx*
Smt3h2-ps	*SNORA17*	*SNORA40*	*SNORA70*	*Spaca5*
Srpx	*Ssx9*	*Ssxa1*	*Ssxb1*	*Ssxb10*
Ssxb2	*Ssxb3*	*Ssxb5*	*Ssxb6*	*Ssxb8*
Ssxb9	*Suv39h1*	*Syn1*	*Syp*	*Sytl5*
Tbc1d25	*Tfe3*	*Timm17b*	*Timp1*	*Tspan7*
U1	*U1*	*U2*	*U2*	*U6*
U6	*U6*	*U6*	*U6*	*U6*
U6	*U6*	*U6*	*U6*	*U6*
U6	*Uba1*	*Usp11*	*Usp27x*	*Usp9x*
Uxt	*Was*	*Wdr13*	*Wdr44*	*Wdr45*
Xk	*Zfp182*	*Zfp300*		

Supplementary Table 4: Genes in the Chromosome 4 locus Awt1.

1110049F12Rik	*1700013G24Rik*	*1700021N21Rik*	*1700029M20Rik*	*1700037C06Rik*
1700095J12Rik	*1810058N05Rik*	*2310026L22Rik*	*2610002D18Rik*	*2610528B01Rik*
4930549C01Rik	*4930555I21Rik*	*6030445D17Rik*	*9130020K20Rik*	*A330049M08Rik*
AB041806	*AL645531.1*	*AL669982.1*	*AL670720.1*	*AL671011.1*
AL671011.2	*AL671173.1*	*AL672076.1*	*AL807764.1*	*Alpl*
Asap3	*BX000694.1*	*C1qa*	*C1qb*	*C1qc*
Camk2n1	*Catsper4*	*Ccdc21*	*Cda*	*Cdc42*
Cela3b	*Clic4*	*Cnksr1*	*Cnr2*	*D4Wsu53e*
Ddost	*E130218I03Rik*	*E2f2*	*Ece1*	*Eif4g3*
Epha8	*Ephb2*	*Extl1*	*Fam43b*	*Fam54b*
Fuca1	*Gale*	*Gm12982*	*Gm12983*	*Gm12984*
Gm12986	*Gm12987*	*Gm12988*	*Gm12989*	*Gm12990*
Gm12991	*Gm13000*	*Gm13001*	*Gm13002*	*Gm13003*
Gm13005	*Gm13006*	*Gm13007*	*Gm13008*	*Gm13009*
Gm13010	*Gm13011*	*Gm13012*	*Gm13013*	*Gm13030*
Gm13131	*Gm13195*	*Gm13250*	*Gm15979*	*Gm16224*
Gm16225	*Gm7534*	*Grhl3*	*Grrp1*	*Hmgcl*
Hnrnpr	*Hp1bp3*	*Hspg2*	*Htr1d*	*Id3*
Il22ra1	*Il28ra*	*Kdm1a*	*Kif17*	*Lactbl1*
Ldlrad2	*Ldlrap1*	*Luzp1*	*Lypla2*	*Man1c1*
Mir700	*Mul1*	*Myom3*	*Nipal3*	*Otud3*
Pafah2	*Paqr7*	*Pdik1l*	*Pink1*	*Pla2g2a*
Pla2g2c	*Pla2g2d*	*Pla2g2e*	*Pla2g2f*	*Pla2g5*
Pnrc2	*Rap1gap*	*Rcan3*	*Rhd*	*Rpl11*
Rpl31-ps10	*Rpl38-ps1*	*Runx3*	*Sepn1*	*Sh2d5*
Sh3bgrl3	*Slc30a2*	*SNORA17*	*Srrm1*	*Srsf10*
Stmn1	*Syf2*	*Tcea3*	*Tceb3*	*Tmem50a*
Tmem57	*Trim63*	*U1*	*U6*	*U6*
U6	*U6*	*Ubxn10*	*Ubxn11*	*Usp48*
Vwa5b1	*Wnt4*	*Zbtb40*	*Zfp46*	*Zfp593*

Supplementary Table 5: Genes in the Chromosome 13 locus Awtq2.

1700024P04Rik	*1700029F12Rik*	*1700119I11Rik*	*4833422C13Rik*	*5330416C01Rik*
7SK	*7SK*	*AC123043.1*	*AC124515.1*	*AC130536.1*
AC131739.1	*AC136976.1*	*AC137692.1*	*AC154225.1*	*AC154271.1*
AC154424.1	*AC154460.1*	*AC154460.2*	*AC160109.1*	*AC161588.1*
Acot12	*Aggf1*	*Ankdd1b*	*Ankra2*	*Ankrd34b*
Ap3b1	*Arsb*	*Atg10*	*Atp6ap1l*	*AW495222*
Bhmt	*Bhmt2*	*Btf3*	*C030017D09Rik*	*Ccnh*
Ckmt2	*Cmya5*	*Col4a3bp*	*Cox7c*	*Crhbp*
Dhfr	*Dmgdh*	*Edil3*	*Enc1*	*F2r*
F2rl1	*F2rl2*	*Fam151b*	*Fam169a*	*Fcho2*
Foxd1	*Gcnt4*	*Gfm2*	*Gm10320*	*Gm11019*
Gm15620	*Gm15622*	*Gm15907*	*Gm16243*	*Gm16318*
Gm17190	*Gm17352*	*Gm17450*	*Gm17622*	*Gm4117*
Gm5453	*Gm6169*	*Gm8526*	*Gm9776*	*Gm9828*
Hapln1	*Hexb*	*Hmgcr*	*Homer1*	*Iqgap2*
Jmy	*Lhfpl2*	*Mrps27*	*Msh3*	*Mtx3*
Nsa2	*Otp*	*Papd4*	*Pde8b*	*Poc5*
Polk	*Ptcd2*	*Rasa1*	*Rasgrf2*	*Rgnef*
RP24-76E2.4	*Rps23*	*S100z*	*Scamp1*	*Serinc5*
SNORA17	*Snora47*	*SNORA68*	*snoU109*	*snoZ39*
Spz1	*Ssbp2*	*Sv2c*	*Tbca*	*Thbs4*
Tmem167	*Tmem171*	*Tmem174*	*Tnpo1*	*U1*
U6	*U6*	*U6*	*Utp15*	*Vcan*
Wdr41	*Xrcc4*	*Zbed3*	*Zcchc9*	*Zfp366*
Zfyve16				
